# Our New York Letter

**Published:** 1888-09

**Authors:** 


### Correspondence.

### OUR NEW YORK LETTER.

ELECTRICAL EXPERIMENTS UPON DOGS—THE PATHOLOGICAL SOCIETY—DEMONSTRATIONS BY MICRO-PHOTOGRAPHS—TUBERCLE AND SYPHILIS—A NEW PRESERVATIVE FOR ANATOMICAL SPECIMENS—SQUIBB ON SULPHUR—THE CONTEST OVER DR. ALONZO CLARK'S WILL—DR. AGNEW'S SUCCESSOR AT THE COLLEGE OF PHYSICIANS AND SURGEONS.

*Editors Atlanta Medical and Surgical Journal:*

Since electricity has come into such general use for lighting the streets of New York, fatal accidents from this source have been quite common. The victims have mostly been linemen, whose business brought them into close quarters with the wires, but several other persons have been killed while walking along the streets. Some very instructive experiments were performed in this connection a few days ago by Mr. Harold P. Brown, electrical engineer, at the school of mines, to illustrate the comparative dangers of high tension continuous and high tension alternating currents. A large black dog was placed in a box covered with wire, and an electrode attached to one front and one hind paw. The resistance was stated to be 15.300 ohms, and the continuous current was used, there being rests between each application through a relay. At 300 volts the animal barked, howled and tried to bite the electrodes. At 400 volts he appeared to become more infuriated. At 500 volts the dog made violent efforts to get out of the box, and Mr. Brown stated that a current like this would kill but for the relay. At 700 volts his exertions to become free increased, accompanied with a noise resembling choking.

At 1,000 volts the electric lamps in connection with the current became luminous, and the dog struggled still more, if that were possible. The continuous current was now changed for the alternating one, and at 330 volts death was produced. A representative of the Society for the Prevention of Cruelty to Animals stopped the proceedings at this stage, and several other dogs waiting in the cellar were prevented from being experimented upon. Mr. Brown said that all currents of 500 volts or more are dangerous to life, and alternating currents were never safe. In time it appears probable that all the electric wires will be buried under ground.

One of the most successful medical organizations here is the Pathological Society, which continues to grow steadily, and is at present in a very flourishing condition. A very interesting feature was an exhibition of micro-photographs illustrating the lesions of acute Bright's disease, which was given by Dr. Francis Delafield at a meeting of this society. In his usual clear and terse manner he describes the kidney changes, and by means of a magic lantern the photographs were thrown upon a large screen. The society by a vote of thanks expressed their appreciation of this beautiful demonstration. Dr. J. J. Griffiths showed specimens of acute phthisis taken from a child who died at the age of about 13 months. The child had suffered from diarrhœa and hemiplegia, with enlarged glands and signs of pulmonary trouble. The specimens were interesting because of the extensive tubercular deposit in the lungs and lymphatics, no tubercles existing in other parts of the body. No cerebral lesions existed to account for the marked hemiplegia, and the epitrochlear glands were enlarged from tubercles and not, as might have been expected, from syphilis. The President, Dr. Wm. P. Northrup, said it had been dogmatically stated that enlarged epitrochlear glands indicated syphilis, but in this case there were no signs of this disease. Dr. Ridlon thought it possible that hereditary syphilis existed in this case, for he had seen many children with a straight history of hereditary syphilis who exhibited tubercular bone lesions. He thought perhaps there was a connection between hereditary syphilis and tubercle in children. Dr. T. M. Prudden gave the formula for a new preserving fluid which he had compounded as follows: Water 1000 c. c. Salt 100 gm. Saltpetre 25 gm. Carbolic acid 5 gm. Glycerine 15 c. c. Amylic or ethylic alcohol 100 c. c. The specimens should be first placed in brine. A piece of intestine, which had been in this fluid nine weeks during warm weather, showed plainly the colors of the fresh tissues. Its principal use is to preserve gross specimens temporarily without changing their color or general appearance.

Dr. J. Lewis Smith read a letter from Dr. Squibb, the Brooklyn chemist, in which the latter doubted the efficacy of burning sulphur as a disinfectant. He said its power would be greatly increased if the fumes were allowed to mingle with water. Many good authorities admit that burning sulphur destroys bacteria, but he said it would not kill the spores.

Dr. Alonzo Clark, who died last September, willed a house to the College of Physicians and Surgeons in trust, and decided that part of the income be devoted to maintain a scholarship for a person studying three years under the faculty to discover new medical facts. Part was to be paid to the graduate writing the best medical essay. The rest was to be used for special lectures. Another bequest of a house was made to the college, to be accepted after his nephews had received the income for 15 years. This will has just been contested, and the judicial decision declares the second bequest void, being contrary to law, and that both fail owing to the defendants not answering.

The vacancy in the chair of ophthalmology at the College of Physicians and Surgeons, caused by Dr. Agnew's death, has been filled by Dr. Hermann Knapp.

Dr. Chas. S. Bull has just been appointed to the chair of ophthalmology at the University Medical College.

A. F.

New York, August 8, 1888.

